# Glutathione depletion triggers actin cytoskeleton changes via actin-binding proteins

**DOI:** 10.1590/1678-4685-GMB-2017-0158

**Published:** 2018-06-04

**Authors:** Nahum Zepeta-Flores, Mahara Valverde, Alejandro Lopez-Saavedra, Emilio Rojas

**Affiliations:** 1 Universidad Nacional Autónoma de México Universidad Nacional Autónoma de México Instituto de Investigaciones Biomédicas Departamento de Medicina Genómica y Toxicología Ambiental MéxicoD.F. Mexico Departamento de Medicina Genómica y Toxicología Ambiental, Instituto de Investigaciones Biomédicas, Universidad Nacional Autónoma de México, México D.F., Mexico; 2 Universidad Nacional Autónoma de México Universidad Nacional Autonóma de México Instituto de Investigaciones Biomédicas Instituto Nacional de Cancerología MéxicoD.F. Mexico Unidad Biomédica de Investigación en Cáncer, Instituto de Investigaciones Biomédicas, Universidad Nacional Autónoma de México, Instituto Nacional de Cancerología, México. D.F., Mexico

**Keywords:** Glutathione, BSO, thymosin β4, gelsolin, profiling

## Abstract

The importance of glutathione (GSH) in alternative cellular roles to the canonically proposed, were analyzed in a model unable to synthesize GSH. Gene expression analysis shows that the regulation of the actin cytoskeleton pathway is strongly impacted by the absence of GSH. To test this hypothesis, we evaluate the effect of GSH depletion via buthionine sulfoximine (5 and 12.5 mM) in human neuroblastoma MSN cells. In the present study, 70% of GSH reduction did not induce reactive oxygen species, lipoperoxidation, or cytotoxicity, which enabled us to evaluate the effect of glutathione in the absence of oxidative stress. The cells with decreasing GSH levels acquired morphology changes that depended on the actin cytoskeleton and not on tubulin. We evaluated the expression of three actin-binding proteins: thymosin β4, profilin and gelsolin, showing a reduced expression, both at gene and protein levels at 24 hours of treatment; however, this suppression disappears after 48 hours of treatment. These changes were sufficient to trigger the co-localization of the three proteins towards cytoplasmic projections. Our data confirm that a decrease in GSH in the absence of oxidative stress can transiently inhibit the actin binding proteins and that this stimulus is sufficient to induce changes in cellular morphology via the actin cytoskeleton.

## Introduction

Gluthathion (GSH) is a tripeptide synthesized in two adenosine triphosphate-dependent steps: the glutamate cysteine ligase (GCL; rate-limiting enzyme in GSH synthesis) forms the dipeptide γ-L-glutamyl-L-cysteine, and then the glutathione synthetase binds a glycine to form GSH or γ-L-glutamyl-L-cysteinyl-glycine (Bains and Shaw, 1997; Dringen, 2000). GSH acts as a redox buffer due to its cysteine sulfhydryl group (-SH); GSH can therefore react directly with radicals through non-enzymatic reactions and constitutes the main barrier against oxidative damage caused by reactive oxygen species (ROS). GSH can also form xenobiotic conjugates through its enzymatic glutathione S-transferase activity and acts as an electron donor in the peroxide reduction catalyzed by the glutathione peroxidase enzyme (GPx) (Camera and Picardo, 2002).

Additionally, GSH and GSH metabolism have been implicated in cancer prevention, progression and treatment response. As a scavenger molecule, GSH can inhibit the action of different molecules through their interaction, including harmful molecules and anticancer drugs (Siddik, 2003; Wang and Lippard, 2005; Franco and Cidlowski, 2009; Chen and Tien Kuo, 2010). Also, GSH levels appear to be reduced after exposure to some xenobiotics (Becker and Soliman, 2009; Pierozan *et al.*, 2016). In the nervous system, GSH is needed for defense against oxidative stress, and alterations in GSH metabolism have been reported in different pathologies including Parkinson’s disease and Alzheimer’s disease (Bains and Shaw, 1997; Ramaekers and Bosman, 2004). Thus, a reduced level of GSH appears to represent an important step in cell destabilization.

In addition to its several functions, data generated in our workgroup on a GCS-2 cell line (a GCL knockout cell line that is unable to synthesize GSH (Shi *et al.*, 2000; Rojas *et al.*, 2003; Valverde *et al.*, 2006) suggested that the reduced level of intracellular GSH (2% of the wild type value of GSH) could generate changes in the expression of several genes. In addition, these cells can survive in the complete absence of GSH if N-acetyl cysteine is provided in the medium; thus, the reducing equivalents provided by GSH, and not GSH itself, protect cells from apoptosis. This means that GCS-2 cells with a severe reduction in GSH produce a metastable state compatible with survival. The results provided by this particular cell model could indicate that cell survival is compatible with low GSH intracellular levels without alterations of redox. In the present study, from the analysis of global expression of the GSC-2 model identifies the role of the absence of GSH in the pathways involved in the remodeling of the actin cytoskeleton and explores the hypothesis in a neuroblastoma model, under intracellular GSH modulation.

This study used MSN neuroblastoma cells to represent an early stage in neuronal development in which cells are pluripotent and retain the capability of expressing multiple neural crest-derived phenotypes (Abemayor and Sidellt, 1989). These cells also appear to be very sensitive to thiols depletion and have been used previously to observe changes in the cytoskeleton (Arias *et al.*, 1993; Ko *et al.*, 1997; Stabel-Burow *et al.*, 1997).

The cytoskeleton is a dynamic system that consists of several filamentous networks that extend from the plasma membrane to the nuclear envelope and interconnect the cell nucleus to the extracellular matrix. Actin polymerizes to form filaments and participates in the generation and maintenance of cell morphology, polarity, endocytosis, intracellular trafficking, contractility, motility and cell division (Ramaekers and Bosman, 2004; Fu *et al.*, 2015). However, actin filaments by themselves are not able to perform the processes involved. Instead, these processes require many proteins, including actin-binding proteins. Actin-binding proteins are responsible for orchestrating rounds of polymerization-depolymerization of the actin filaments. Our work focuses on three of the relevant functions of the actin binding proteins in disease development: thymosin β4, gelsolin and profilin (Winder and Ayscough, 2005; Lee *et al.*, 2013; Cui *et al.*, 2016).

Thymosin β4 is a monomer-sequestering protein that maintains a large pool of actin that allows rapid filament growth, clamps ATP-actin, and prevents its incorporation into filaments (Dedova *et al.*, 2006). Gelsolin is a capping and severing protein that controls filament length by capping the barbed end, blocking the addition of new monomers and severing actin filaments to increase actin dynamics (Sun *et al.*, 1999). Finally, profilin is also a monomer-sequestering protein but is involved in binding to ADP-actin to promote the nucleotide exchange (ADP for ATP) and facilitate new rounds of polymerization (Hertzog *et al.*, 2004; Birbach, 2008).

Abnormalities in these essential cell components often result in disease (Ramaekers and Bosman, 2004). Studies have shown that the actin cytoskeleton and the actin-binding proteins participate in many processes related to carcinogenesis, such as invasion, metastasis and the epithelial-mesenchymal transition (Kedrin *et al.*, 2007; Lorente *et al.*, 2014; Cui *et al.*, 2016). Moreover, alterations in cytoskeletal components have been implicated in the progression of some neurodegenerative disorders (McMurray, 2000; Ying *et al.*, 2004).

Given the essential roles of the actin cytoskeleton and GSH in physiological and pathological processes, the aim of the present study was to confirm the hypothesis generated by the model GCS-2 in a scenario of GSH depletion in the regulation and remodeling of the actin cytoskeleton in human MSN neuroblastoma cells.

## Materials and Methods

### GCS-2 cell model

#### Cell culture

All studies used M15 complete medium, “knockout” DMEM supplemented with 15% embryonic stem cell-qualified FBS, 2 mM glutamine, 0.1 mM β-mercaptoethanol (BME), 100 units/mL of penicillin, and 100 μg/mL of streptomycin (all from GIBCO/BRL, Carlsbad, CA, USA, except BME, from Sigma-Aldrich, St. Louis, MO, USA). Mouse blastocyst cells derived from GCS ^-/-^ and the ^+/+^ BDC1 mice were obtained. γGCS-deficient cells (GCS-2) were maintained in the above medium with 2.5 mM GSH (Sigma-Aldrich) and changed with fresh GSH containing medium daily. In the experiments involving GSH withdrawal, cells were washed twice with 1X PBS (GIBCO/BRL) and replaced with complete medium without supplemental GSH. Cultures were maintained at 37 °C in humidified incubators containing 5% CO_2_. Intracellular GSH levels in γGCS-deficient cells grown in the presence or absence of GSH were determined using HPLC/EC detection as described by Kleinman and Riche Jr (1995). Viability and cell number were determined using trypan blue exclusion staining (Sigma-Aldrich). Briefly, aliquots of 2.5 x 10^5^ cells/mL were mixed with the trypan blue, and cells were examined by light microscopy. The results represent the average of three independent experiments with duplicate determinations.

#### cRNA preparation

We isolated total RNA using Trizol reagent (Invitrogen, Carlsbad, CA, USA) and purified the RNeasy Total RNA Isolation Kit (Qiagen, Hilden, Germany). The SuperScript Choice system (Invitrogen) was used to synthesize double-stranded cDNA. Phase Lock Gels-phenol and chloroform extractions (Eppendorf, Hamburg, Germany) were used to clean up the cDNA template. We then generated biotin-labeled cRNA from this template using a BIOARRAY HIGHYIELD RNA Transcript Labeling Kit (Enzo Life Sciences, Farmingdale, NY, USA). *In vitro* transcription products were purified using RNeasy spin columns (Qiagen) and were quantified by spectrophotometric analysis. After the purification, the cRNA was fragmented using the standard procedure by Affymetrix to obtain a distribution of RNA fragments sized from approximately 35 to 200 bases. Fragmented RNA was checked with agarose gel electrophoresis.

#### Microarray analysis

A hybridization cocktail was prepared as recommended by Affymetrix, containing 0.05 μg/μL fragmented cRNA, 50 pM control oligonucleotide B2, 1.5, 5, 25 and 100 pM eukaryotic hybridization controls with *bioB, bioC, bioD* and *cre* genes, respectively, 0.1 mg/mL herring sperm DNA, 0.5 mg/ml acetylated BSA and 1X hybridization buffer. This hybridization cocktail was heated to 99 °C for 5 min and then used to fill the probe array cartridge. Hybridization was performed for 16 h with a rotation of 60 rpm in a rotisserie oven at 4 5°C.

After 16 h of hybridization, the hybridization cocktail was removed from the probe array, and the array was filled with non-stringent wash buffer. The GeneChip® Fluidics Station 400 (Affymetrix, Inc., Santa Clara, CA, USA) operated using Microarray Suite was used to wash and stain the probe arrays. We followed the manufacturer’s single stain protocol for eukaryotic targets. Arrays were washed twice and stained with a 10 μg/L streptavidin phycoerythrin solution. After staining, a final wash with non-stringent buffer was performed, and the arrays were scanned.

#### Data analysis

Image quantification, background subtraction and scaling were carried out with dChip software (Harvard, Boston, MA, USA) with 100% recall between control and lower GSH level chips and *p*<0.05 for the statistical algorithm (Li and Wong, 2001). DAVID Bioinformatics resources 6.8 was used to analyze the impacted pathways (Huang *et al.*, 2009).

### MSN cell model

Adherent human neuroblastoma MSN cells were grown in monolayers (Reynolds *et al.*, 1986; Ramos-Espinosa *et al.*, 2012). The cells were cultured in RPMI 1640 medium (Sigma-Aldrich) supplemented with 10% fetal bovine serum (Gibco, Life Technologies Corporation, Grand Island, NY, USA), 1% antibiotic-antimycotic (penicillin-streptomycin-amphotericin) (Gibco), 1% MEM non-essential amino acids (Gibco) and 1% QSN (glutamine-serine-asparagine) in tissue culture dishes in a humidified incubator under 95% air and a 5% CO_2_ atmosphere at 37 °C. Cells were subcultured at a density of 1x10^6^ cells per dish and harvested by gently pipetting.

#### GSH depletion (BSO treatments)

A total of 1x10^6^ MSN cells were seeded into a 100 mm tissue culture dish with 7 mL of supplemented RPMI 1640 medium. After 72 h, the culture was gently washed with PBS buffer, and 10 mL of supplemented RPMI 1640 medium was added. Buthionine sulfoximine (BSO, L-Buthionine-sulfoximine Sigma-Aldrich) treatments were administered at final concentrations of 0, 5, 12.5, 25 and 50 mM.

#### Cell viability

Cell viability was measured using the dual stain fluorescein diacetate (FDA)/ethidium bromide (BrEt) method as previously described (Hartmann and Speit, 1997). Briefly, the cells were mixed with a fluorochrome solution containing 0.02 μg/mL Et-Br and 0.015 μg/mL FDA (Sigma-Aldrich). The cells were then analyzed under an Olympus BX-60 fluorescence microscope with a UM61002 filter (Olympus, Tokyo, Japan). One hundred randomly chosen cells were evaluated per condition.

#### Reduced glutathione quantification

We used o-phthalaldehyde (OPT) (Sigma-Aldrich) as the fluorescent reagent to quantify the level of reduced glutathione due its specificity for GSH. A total of 100 μL of cells in PBS supplemented with protease inhibitors and 100 μL of meta-phosphoric acid (Sigma-Aldrich) precipitating reagent (1.67 g meta-phosphoric acid, 0.2 g EDTA (AMRESCO, Solon, OH, USA) and 30 g NaCl in 100 mL of distilled water) were added to a 0.6 mL Eppendorf tube, vortexed and centrifuged. The supernatant was decanted and frozen at -70 °C prior to further quantification. A total of 50 μL of the supernatant was added to a 1.5 mL Eppendorf tube with 1 mL of GSH buffer (0.1 M NaH_2_PO_4_ and 0.005 M EDTA, pH 8.0). Then, 50 μL of OPT (1mg/mL in methanol) was added to obtain a GSH-fluorescent conjugate. Next, 200 μL of the mixture from each Eppendorf tube was plated in an opaque 96-well plate and incubated for 15 min in the dark. The fluorescence was read in a BioTek FLx800 Fluorescence Microplate Reader (Winooski, VT, USA) with the emission set at 420 nm and the excitation set at 350 nm (Browne and Armstrong, 1998).

#### Reactive Oxygen Species (ROS)

ROS were determined with a modified fluorometric assay (Lee *et al.*, 2003), which employs dihydrorhodamine 123 (DHR; Calbiochem-EMD Chemicals Inc. San Diego, CA, USA) as the probe. When DHR is oxidized by H_2_O_2_ in the presence of peroxidases, it produces the fluorescent compound rhodamine 123. Briefly, 100 μL of cells in PBS supplemented with protease inhibitors were centrifuged at 1,200 rpm for 5 min. Then, the supernatant was discarded, and 180 μL of buffer A (140 mM NaCl, 5 mM KCl, 0.8 mM MgSO_4_•7H_2_O, 1.8 mM CaCl_2_, 5 mM glucose and 15 mM HEPES, Sigma-Aldrich) and 20 μL of DHR (1 μM) were added and incubated at 37 °C for 2 min. The fluorescent product rhodamine 123 was measured using a spectrophotometer at 505 nm and interpolated in a curve of rhodamine 123.

#### Lipid Peroxidation (Lpx)

The thiobarbituric acid method was used to measure the concentration of malondialdehyde (MDA) (Bouaïcha and Maatouk, 2004). Briefly, 100 μL of cells in PBS supplemented with protease inhibitors and 100 μL of trichloroacetic acid (10% w/v) (Avantor Performance Materials Inc. Center Valley, PA, USA) were added to 1.5 mL Eppendorf tubes and centrifuged at 3,000 x *g* for 10 min. The supernatant was added to 1 mL of the thiobarbituric acid reagent (0.375%) (ICN Biomedicals Inc. Aurora, OH, USA), and the mixture was heated at 92 °C for 45 min. The absorbance of the thiobarbituric acid-MDA complex was measured at 532 nm using an ELISA spectrophotometer (Model 550 microplate reader, Bio-Rad, Hercules, Californa, USA). The data were interpolated onto a concentration curve of MDA (1,1,3,3-tetraethoxypropane) ranging from 0 to 10 nM.

#### Reverse transcriptase-polymerase chain reaction

Total RNA was isolated using TRIzol Reagent (Invitrogen) following the manufacturer’s protocol. The RNA quantity and purity were determined spectrophotometrically. The reverse transcriptase-polymerase chain reactions (RT-PCR) were performed using the Access RT-PCR System (Promega, MADISON, WI, USA) according to the manufacturer’s recommendations. The RT-PCR products were loaded onto a 3% agarose gel, and the mRNA levels were analyzed using the Kodak 1D v3.5.3 software. The following primers were used:

Actin (forward: catcatgaagtgtgacgtgg; reverse: atactcctgcttgctgatcc),

Thymosin β4 (forward: tgaacaggagaagcaagcag; reverse: tagacagatgggaaaggcag),

Gelsolin (forward: acggctgaaggacaagaaga; reverse: ttccaacccagacaaagacc),

Profilin (forward: ggaggcggattgaataagaag; reverse: ccatcaccctgcattgctaa) and

Ribosomal Protein L32 (forward: aagaagttcatccggcaccag; reverse: gcgatctcggcacagtaagat).

#### Western blot analysis

MSN cells were lysed in RIPA buffer (150 mM NaCl, 0.1% Triton X-100, 0.5% sodium deoxycholate, 0.1% sodium dodecyl sulfate and 50 mM Tris-HCl, pH 8.0) with a protease inhibitor mixture and then centrifuged. Total proteins were quantified using a bicinchoninic acid kit (Thermo-Fisher Scientific Inc., Rockford, IL, USA). Equal amounts of protein were loaded onto a polyacrylamide gel and then transferred to a nitrocellulose or polyvinylidene fluoride membrane as required (Schägger, 2006). The membrane was blocked with 5% nonfat milk and 1% albumin in TBS and then sequentially incubated with a primary antibody and horseradish peroxidase-conjugated secondary antibody. The primary antibodies employed were goat polyclonal anti-Thymosin β4 (1:2000), mouse monoclonal anti-Gelsolin (1:2000), rabbit polyclonal anti-Profilin 1/2 (1:2000), and mouse monoclonal anti-β Tubulin (1:2000) (all Santa Cruz Biotechnology, Santa Cruz, CA, USA); the antibodies were dissolved in blocking solution and incubated at 37 °C for 1 h. Then, the membranes were washed with TBS-0.1% Tween-20 and incubated with the secondary antibody. The secondary antibodies employed were HRP-conjugated goat anti-rabbit, HRP-conjugated rabbit anti-goat and HRP-conjugated goat anti-mouse, all supplied by Zymed (San Francisco, CA, USA). The antibodies were dissolved in blocking solution and incubated at 37 °C for 1 h. The bands were visualized using the Amersham ECL Western blotting detection reagents according to the manufacturer’s guidelines (GE Healthcare, Piscataway, NJ. USA).

#### Immunofluorescence

A total of 10^5^ MSN cells were seeded onto coverslips coated with 0.1% gelatin in a 12-well plate and incubated for 72 h or until sub-confluent. Then, the culture was gently washed with PBS buffer, the medium was renewed and the 5 and 12.5 mM BSO treatments were added. After 24 or 48 h of BSO treatment, the media was removed and the MSN cells were fixed with 4% paraformaldehyde and permeabilized with PBS-0.2% Triton X-100. Prior to antibody incubation, the samples were blocked with 1% albumin and 5% fetal bovine serum in PBS. The primary antibodies employed were mouse anti-β-Tubulin (1:50; Invitrogen), goat polyclonal anti-Thymosin β4 (1:50, Santa Cruz Biotechnology), mouse monoclonal anti-Gelsolin (1:50, Santa Cruz Biotechnology) and rabbit polyclonal anti-Profilin 1/2 (1:50, Santa Cruz Biotechnology). The antibodies were dissolved in blocking solution and incubated at 37 °C for 1 h. Then, the coverslips were washed three times with PBS-0.2% Triton X-100 for 5 min. The secondary antibodies employed were FITC-goat anti-mouse (1:100, Invitrogen), FITC-goat anti-rabbit (1:100, Invitrogen) and FITC-bovine anti-goat (1:100, Santa Cruz Biotechnology). Alexa Fluor® 594 Phalloidin (1:50, Invitrogen) was also employed to stain the actin filament, and 300 nM 4’,6-diamidino-2-phenylindole (DAPI, Sigma-Aldrich) was used to counterstain the cell nuclei. All of the secondary antibodies were dissolved in blocking solution. The cells were incubated with the secondary antibodies and Alexa Fluor® 594 Phalloidin at 37 °C for 1 h; DAPI was added during the last 5 min of the secondary antibody incubation. Finally, the coverslips were washed three times with PBS-0.2% Triton X-100 for 5 min, embedded in Dako Fluorescent Mounting Medium (Dako North America, Inc., Carpinteria, CA, USA) and mounted onto slides. The slides were examined using an Axio Observer inverted microscope (Carl Zeiss, Oberkochen, Germany) coupled to a confocal laser scanning LSM 710 DUO from Carl Zeiss (Plan Apochromat 40X/1.3 oil objective). Images were acquired using the ZEN 2008 software (Carl Zeiss) and analyzed using the Fiji image processing package, which is distributed by ImageJ (Schindelin *et al.*, 2012).

#### Statistical analysis

The results are expressed as the percentage ± standard error (SE). Statistical significance was analyzed using one-way ANOVA and Student’s *t*-test, and *p*-values < 0.05 were considered statistically significant. All analyses were performed using the statistical software SigmaStat (Systat Software Inc., San Jose, CA, USA), and histograms were generated using the SigmaPlot software. We also performed a multiple linear regression using the SigmaStat software to determine whether the BSO treatments were related to the changes in cell viability, ROS and lipid peroxidation.

## Results

### Expression of actin cytoskeleton genes in cells unable to synthesize GSH-GCS-2

Global analysis of molecular functions in cells unable to synthesize GSH, obtained by the gene expression patterns and the number of genes involved with their respective *p*-value is presented in Table 1.

**Table 1:** Analysis of molecular functions impacted by the lack of GSH in mouse embryonic cells GCS-2 by DAVID Bioinformatics Resources 6.8.

Term	Count	%	*p* value	Fold Enrichment	Benjamini	FDR
MF00107: Kinase	86	5.77956989	6.68968E-07	1.72558967	0.00013980	0.00084452
MF00108: Protein kinase	68	4.56989247	9.29454E-06	1.73700140	0.00097081	0.01173303
MF00126: Dehydrogenase	32	2.15053763	0.00028276	2.00021197	0.01950907	0.35638051
MF00123: Oxidoreductase	71	4.77150537	0.00059677	1.49772870	0.02464379	0.75077381
**MF00261: Actin binding cytoskeletal protein**	**40**	**2.68817204**	**0.00280201**	**1.62442364**	**0.07068307**	**3.48030055**
**MF00091: Cytoskeletal protein**	**73**	**4.90591397**	**0.00763658**	**1.34716657**	**0.10130483**	**9.22408705**
MF00071: Translation factor	17	1.14247311	0.00281380	2.28303897	0.06333992	3.49470003
MF00166: Isomerase	21	1.41129032	0.00376944	2.00593441	0.07589561	4.65577076

DAVID Bioinformatics Resources 6.8;Term: Enrichment terms associated with the gene list; Count: Gene involved in the term; % : involved genes / total genes; *p*-value: modified Fisher exact *p*-value; Fold Enrichment: To globally correct enrichment*p*-values of individual term members.; Benjamini and FDR: To globally correct enrichment *p*-values to control family-wide false discovery rate under certain rate.

Expression analysis showed that the actin cytoskeleton pathway is largely impacted by GSH absence and that actin binding proteins, such as cofilin 1 and 2, fascin homolog and gelsolin, were overexpressed. In contrast, the thymosin B4, profilin and capping protein muscle z-line beta alpha-2 were underexpressed (Table 2).

**Table 2:** Analysis of gene expression involved in actin cytoskeleton pathway in mouse embryonic cells GCS-2 by DAVID Bioinformatics Resources 6.8.

Unigene ID	Gene Symbol	Name of the gene	Fold Change (log2)
Mm.261329	Myl12a	Myosin	-7.7
Mm.2647	Pfn1	Profilin	-5.16
Mm.97858	Kif1B	Kinesin family member 1B	-4.04
Mm.142729	Tmsb4	Thymosin beta 4	-2.9
Mm.25321	Nudcd3	NudC domain containing 3	-1.92
Mm.21687	Limd2	Lim domain containing 2	-1.91
Mm.392504	Capza2	Capping protein muscle Z-line beta alpha 2	-1.78
Mm.253564	Actn1	Actinin, alpha 1	-1.63
Mm.441340	Kif6	Kinesin family member 6	-1.55
Mm.329322	Fhod3	Formin homolog 2 containing 3	-1.41
Mm.52297	Fnbp1	Formin binding protein 1	-1.41
Mm.428571	Septin 11	Septin II	-1.39
Mm.272460	Gabarap	Gamma.aminobutyric acid receptor associated protein	-1.28
Mm.143877	Mapre1	Microtubule associated protein RP/EB family 1	-1.25
Mm.157770	Cnn2	Calponin 2	1.01
Mm.28357	Map1lc3b	Microtubule associated protein 1 light chain 3 beta	1.2
Mm.99996	Kif1c	Kinesin Family member 1c	1.23
Mm.478285	Dcin5	Dynactin 5	1.26
Mm.278357	Klc1	Kinesin light chain 1	1.26
Mm.276826	Cfl2	Cofilin 2	1.27
Mm.295284	Stom	Stomatin	1.27
Mm.276504	Nudcd2	NudC domain containing 2	1.28
Mm.40068	Tubb3	Tubulin beta 3 Class III	1.3
Mm.7688	Kif3c	Kinesin Family member 3c	1.32
Mm.258986	Mark2	Microtubule affinity regulating kinase 2	1.34
Mm.299774	Jup	Junction plakoglobin	1.34
Mm.272368	Crip1	Cystein-Rich protein 1	1.38
Mm.249479	Dync1i2	Dynein cytoplasmic 1 intermediate chain2	1.38
Mm.5567	Palim1	PDZ and Lim domain 1 (elfin)	1.42
Mm.27063	Trip6	Thyroid hormone receptor interactor 6	1.44
Mm.205601	Cttn	Cortactin	1.48
Mm.30010	Arpc1b	Actin related protein 2/3 complex subunit 1B	1.49
Mm.273538	Tubb5	Tubulin beta 5 class1	1.49
Mm.392113	Tuba1b	Tubulin alpha 1B	1.56
Mm.20829	Emp3	Epithelial membrane protein3	1.6
Mm.271967	Lasp1	Lim and SH3 protein 1	1.61
Mm.208601	Tln1	Talin 1	1.62
Mm.289306	Arpc4	Actin related protein 2/3 complex subunit 4	1.64
Mm.289106	Add1	Aduccin 1 (alpha)	1.64
Mm.6919	Dctn1	Dynactin 1	1.65
Mm.238285	Ketd10	Potassium channel tetramerisation	1.74
Mm.38450	Sept 9	Septin 9	1.88
Mm.21109	Gsn	Gelsolin	1.93
Mm.271711	Tagln2	Transgelin 2	2.11
Mm.329655	Cfl1	Cofilin 1	2.27
Mm.289707	Fscn	Fascin homolog1	2.44
Mm.288974	Arpc5	Actin related protein 2/3 complex subunit 5	2.91
Mm.1287	Mapt	Microtubule-associated protein TAU	3.08
Mm.275648	Pdlim7	PDZ and Lim domain 7	4.18
Mm.441431	Syn2	Synapsin II	4.64
Mm.371777	Pmp2	Peripheral myelin protein 2	5.34
Mm.218624	Sh3yl1	SH3 domain YSC-like 1	13.25

### Depletion of intracellular GSH levels

**Figure 1:**
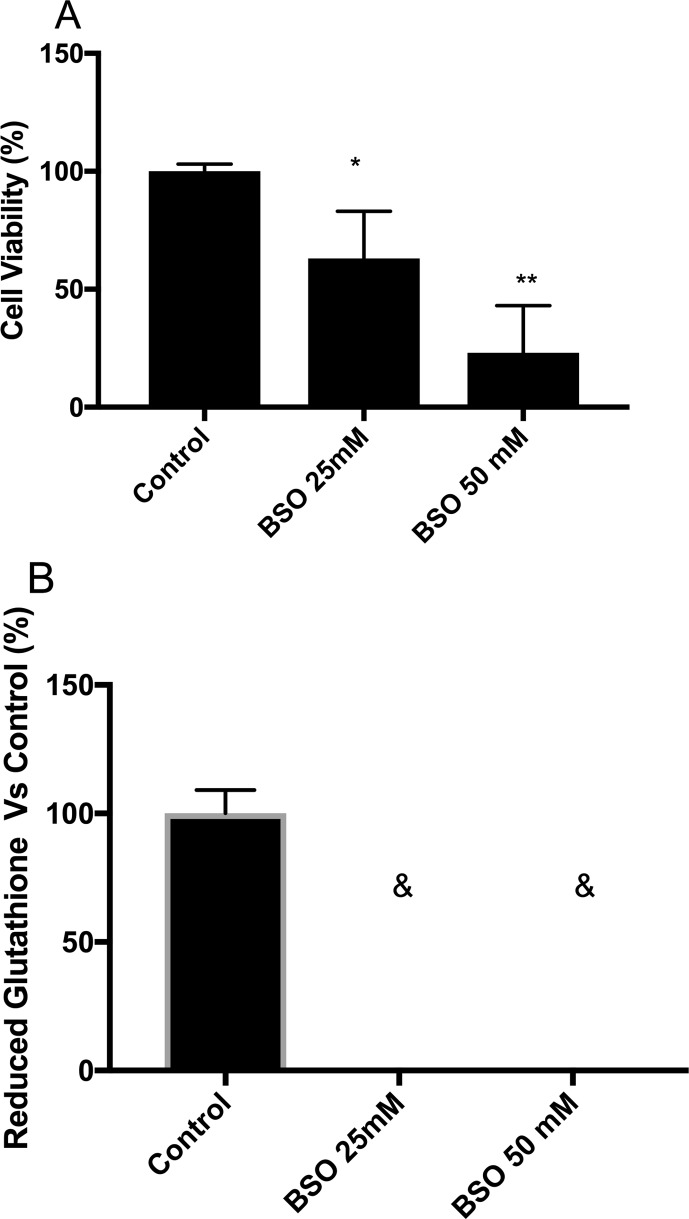
Maximum depletion of intracellular GSH levels generate effects on survival. (A) Cell viability of MSN cells treated with high concentrations of buthionine sulfoximine, BSO, (25 and 50 mM) for 24 h assessed by fluorescein diacetate/ethidium bromide (FDA/EtBr) dual fluorochrome staining. (B) Corresponding intracellular GSH levels of MSN cells treated with BSO (&: not detected) determined fluorometrically (estimated limit of detection for the fluorometric method was 0.31 nmol of GSH/mg of protein). Statistical significance was determined by Student’s *t*-test. *p<0.05, **p<0.01. Experiments were performed three times.

To achieve the maximum GSH depletion in the shortest amount of time, MSN cells were treated with 25 and 50 mM BSO for 24 h. We found significantly decreased cell viability and GSH levels at both concentrations (Figure 1A and 1B, respectively). Therefore, we tested lower BSO concentrations (5 and 12.5 mM) without a cell viability effect (Table 3). The 5 and 12.5 mM BSO treatments for 24 h could decrease the GSH level to approximately 70% compared with the control (Table 3). Therefore, we used these conditions for the assays. After we set up the working BSO concentrations, we evaluated the cell viability and GSH level following treatment for 48 h. The BSO treatments did not affect the cell viability of the MSN cells (Table 3), and the intracellular GSH level was decreased to approximately 80% compared with the control (Table 3).

**Table 3:** Absence of oxidative stress under GSH depletion.

Hours^a^	Treatment	Cell viability^b^,^c^	GSH ^b^,^d^	ROS^b^,^e^	LPx^b^,^f^
	Control	100.00 ± 4.82	100.00 ± 9.05	100.00 ± 6.95	100.00 ± 14.02
24	BSO 5 mM	105.40 ± 2.88	31.33 ± 6.03*	106.75 ± 10.03	82.21 ± 22.06
	BSO 12.5 mM	102.35 ± 0.36	23.21 ± 6.23*	100.91 ± 12.87	104.26 ± 19.08
	Control	100.00 ± 0.74	100.00 ± 6.05	100.00 ± 6.95	100.00 ± 14.02
48	BSO 5 mM	92.21 ± 5.07	15.45 ± 6.10*	109.50 ± 9.35	103.61 ± 19.59
	BSO 12.5 mM	89.14 ± 0.36	18.21 ± 5.09*	113.06 ± 13.96	128.81 ± 19.74

a Length of treatment in hours.

b Data are expressed as the percentage with respect to controls ± standard error.

c Cell viability after BSO treatments assessed by FDA/EtBr dual fluorochrome stain.

d GSH values determined by o-phthalaldehyde (OPT) method after BSO treatment.

e ROS: reactive oxygen species; ROS were measured by the generation of rhodamine 123 in MSN cells after BSO treatment. ROS positive control (Cadmium chloride 50 μM, 2 h): 138.29 ± 13.55.

f LPx: Lipid peroxidation; LPx level assessed with the TBARS method using a MDA curve in MSN cells after treatment with BSO. Experiments were performed three to seven times.

* Student’s *t*-test; *p*<0.01 versus control.

### Absence of oxidative stress under GSH depletion

The redox state evaluation of MSN cells after BSO treatment was determined by measuring ROS levels and the end products of Lpx. It is important to emphasize that our aim was to decrease intracellular GSH levels without initiating an oxidative stress state to establish the effect trigger by GSH depletion on the regulation of the actin cytoskeleton. ROS were measured by the generation of rhodamine 123 in MSN cells treated with 5 and 12.5 mM BSO for 24 and 48 h. No changes in the ROS level were found compared to the control condition, suggesting that the depletion of the intracellular GSH level did not induce ROS generation (Table 3). Additionally, we measured Lpx end products and found that MSN cells treated with 5 and 12.5 mM BSO for 24 h were unable to generate Lpx end products; in contrast, a slight increase was observed after the 48 h treatment with 12.5 mM BSO. To ensure that the loss of GSH did not induce oxidative stress, we conducted a multiple linear regression analysis to evaluate the influence of ROS and Lpx due to GSH depletion by BSO treatment for cell viability (Table 4). Neither ROS nor lipid peroxidation appeared to be necessary to predict cell viability. Thus, the detected ROS and Lpx levels did not affect cell viability, and non-oxidative stress generation could be inferred.

**Table 4:** Multivariate analysis of oxidative stress markers with cell viability.

Multiple linear regression	Coefficient	Standard Error	*p*	VIF^a^
Constant	139.162	50.107	0.069	
ROS^b^	-0.222	0.568	0.722	2.425
LPx^c^	-0.153	0.118	0.284	2.425

a VIF: variance inflation factor;

b ROS: reactive oxygen species;

c LPx: lipid peroxidation. Multiple linear regression model uses a set of independent variables (ROS and LPx) to explain influences on the dependent variable (cell viability). In this case, the analysis shows that none of the independent variables appear necessary to predict cell viability, which means that the detected ROS and Lpx levels do not affect cell viability.

### Glutathione depletion triggers changes in the cell shape

**Figure 2:**
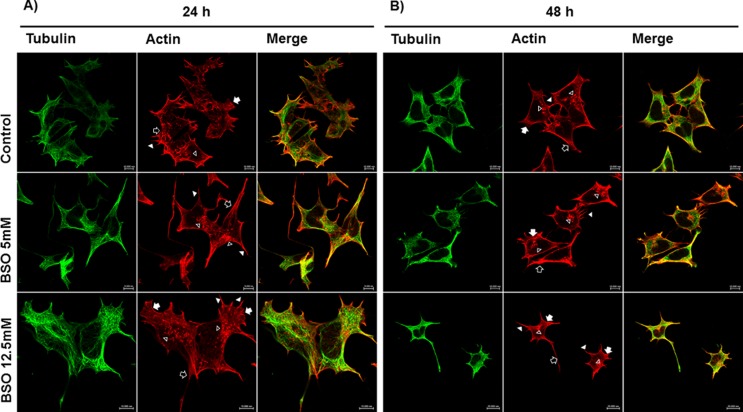
Comparative morphological changes induced by 24 and 48 h BSO treatments. Confocal microscopy images of MSN cells showing that the 48 h BSO treatments caused more drastic morphological changes compared to the 24 h BSO treatments. Panel A corresponds to Control, BSO 5 mM and BSO 12.5 mM 24 h treatments; Panel B corresponds to Control, BSO 5 mM and BSO 12.5 mM 48 h treatments. Microtubules were visualized by staining with FITC-labeled β-Tubulin (mouse anti-β-tubulin/FITC-goat anti-mouse), and actin filaments were visualized by staining with Alexa Fluor® 594 Phalloidin-labeled F-actin. The bar represents 10 μm 40X. Arrow: lamellipodia; arrowhead: filopodia; empty arrow: cone shape; empty arrowhead: cytoplasmic projections and dot-shaped structures.

Confocal microscopy images showed drastic morphological changes in the MSN cells when GSH was depleted by the BSO treatments (Figure 2). After 24 h of GSH depletion, the actin confocal images showed that the control cells displayed the characteristic cell shape and were polarized. Lamellipodia (arrow) with some cytoplasmic projections or filopodia (arrowhead) were observed at one end, whereas a cone shape (empty arrow) with a few cytoplasmic projections and dot-shaped structures (empty arrowhead) that appeared to be focal adhesions was observed at the other end. In the confocal images of control cells the microtubules were clearly resolved as long fibers that were distributed throughout the cytoplasm to the cell periphery and delimited the space occupied by the nucleus. Actin confocal images following the 5 mM BSO treatment showed the loss of the characteristic cell shape and changes in cell polarity, including large cytoplasmic projections along the cell surface, the presence of both lamellipodia (arrow) and filopodia (arrowhead) and dot-shaped structures (empty arrowhead) near the projections. In contrast, the microtubules showed the same characteristics and distribution as the control treatment. Following the 12.5 mM BSO treatment, the actin confocal images showed drastic changes in cell polarity, with the presence of lamellipodia (arrow), many filopodia (arrowhead) and several dot-shaped structures (empty arrowhead). A large filopodium that resembled a neurite (in this case an axon) was observed at one end of the cell. Conversely, the microtubules remained unchanged in structure and organization. The microtubules in both the control and GSH-depleted conditions showed changes in distribution that corresponded to the changes in cell shape due to the actin cytoskeletal rearrangements (Figure 2A).

After the 48 h treatments, the actin filaments and microtubules in the MSN control cells showed a similar pattern to that described following the 24 h control treatment. In the confocal images of the 48 h 5 mM BSO treatment, the loss of the characteristic cell shape present in the MSN control cells was evident, with dramatic polarity changes, retraction of the cytoplasm resulting in a round shape, cytoplasmic projections (filopodia, arrowhead) that extended beyond the leading edge of the membrane and the marked presence of dot-shaped structures (empty arrowhead). No changes were observed in the microtubules. After the 48 h 12.5 mM BSO treatment, the MSN cells showed changes similar to those observed after the 48 h 5 mM BSO treatment: the cytoplasm retracted and became rounded, filopodia (arrowhead) were present along the cell periphery and in some cells a neurite axon-like structure was very evident and gave the cells a neuron-like shape (empty arrow). No important changes were evident in the structure or the organization of the microtubules, however they exhibited changes in distribution corresponding to the actin cytoskeletal rearrangements and the changes in cell shape (Figure 2B).

### Comparison of cell shapes

**Figure 3:**
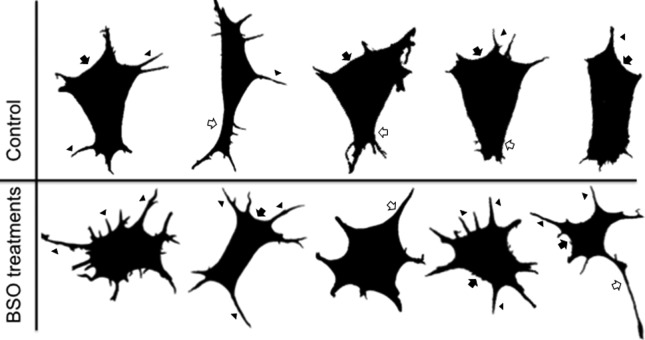
Comparative chart of the predominant cell shapes in 25 cells in the control vs. BSO treatments. Using the image processing package Fiji (distributed by ImageJ), we obtained representative images of the predominant cell shapes in the control conditions (upper panel) and BSO treatments (lower panel). Arrow: lamellipodia; arrowhead: filopodia; empty arrowhead: cytoplasmic projections and dot-shaped structures.

To visualize the changes in cell shape in the confocal images we used the image processing package Fiji (distributed by ImageJ) to isolate 25 images of the predominant cell shapes in the control and BSO treatments. For this, we used the actin confocal images. Figure 3 shows the predominant cell shapes in the control conditions (24 and 48 h, upper panel). In these images, the cells are polarized, one end has a lamellipodium (arrow) with some cytoplasmic projections or filopodia (arrowhead), and the far end has a cone shape (empty arrow) with a few filopodia (arrowhead). In contrast, the different BSO treatments (lower panel) resulted in the constant (approximately 70% of the cells) and very evident loss of the characteristic cell shape, dramatic changes in polarity, more evident cytoplasmic projections, either lamellipodia (arrow) or filopodia (arrowhead) and in some cases the filopodia resembles an axon (empty arrow) and gives the cell a neuron-like cell shape (lower panel).

### Gene expression levels of actin-binding proteins under GSH depletion

**Figure 4:**
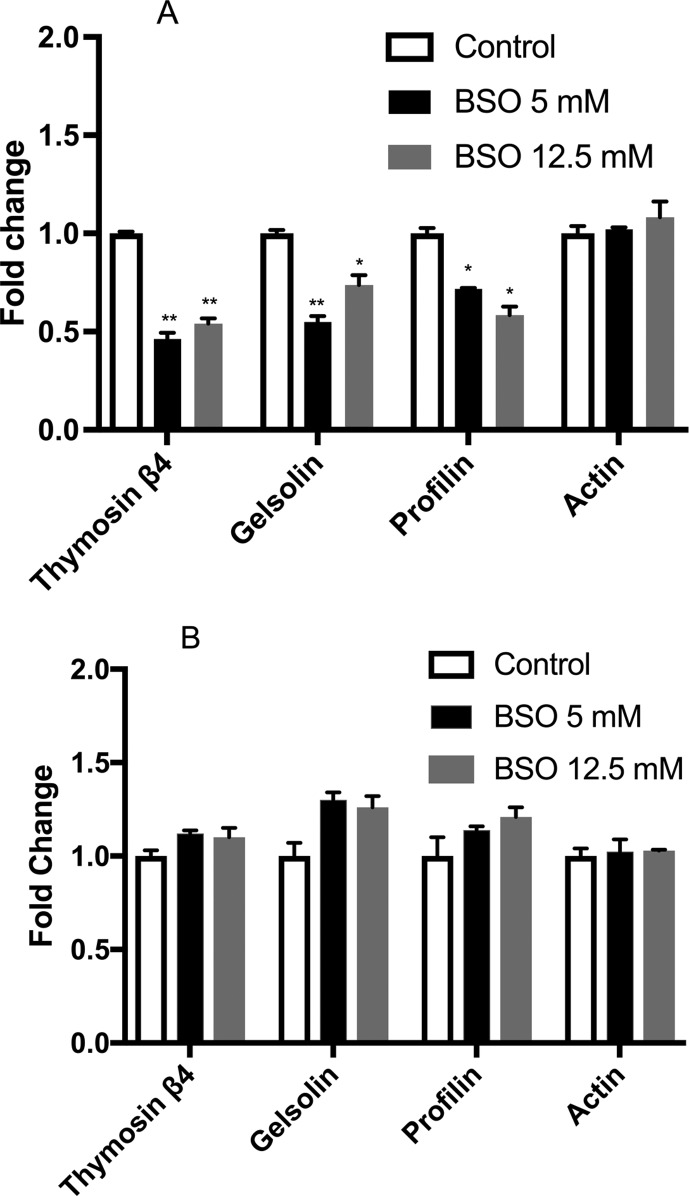
Gene expression. (A) Gene expression in MSN cells treated with 5 and 12.5 mM BSO for 24 h (black and grey bars, respectively). (B) Gene expression in MSN cells treated with 5 and 12.5 mM BSO for 48 h (black and grey bars, respectively). The bars represent fold changes versus the control (open bars); all control values are set to 1. All data were previously normalized to the housekeeping gene *RPL32*. Statistical significance was determined by Student’s *t*-test. *p<0.05. Experiments were performed three times.

Gene expression of the actin-binding proteins thymosin β4, gelsolin, profilin and actin was evaluated after treatment with 5 and 12.5 mM BSO for 24 and 48 h. The GSH depletion for 24 h induced an important decrease in the expression of thymosin β4, gelsolin and profiling (Figure 4A). However, 48 h of GSH depletion show that this sub-expression is lost (Figure 4B).

### Protein expression levels of actin-binding proteins under GSH depletion

**Figure 5:**
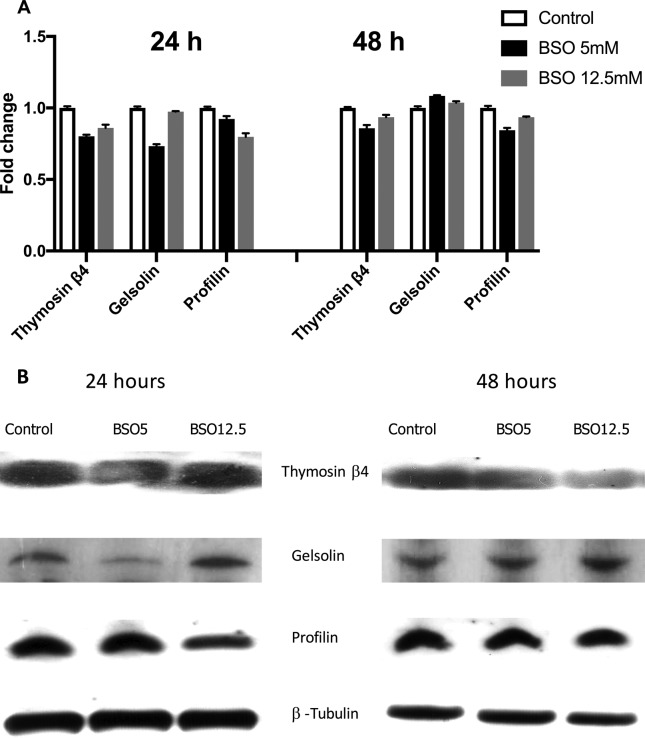
Protein expression. (A) Protein expression in MSN cells treated with 5 and 12.5 mM BSO for 24 h. and 48 h (black and grey bars, respectively). (B) Representative western blots of MSN cells treated with 5 and 12.5 mM BSO for 24 h. and 48 h (black and grey bars, respectively). The bars represent fold changes versus the control (open bars); all control values are set to 1. All data were previously normalized to the β-tubulin. Experiments were performed three times.

The protein expression of thymosin β4, gelsolin, profilin and actin were evaluated after treatment with 5 and 12.5 mM BSO for 24 and 48 h. Analysis showed that gelsolin and thymosin β4 had a 20% reduction in their expression at 24 h after 5 mM treatment. For the treatment with 12.5 mM, however, we only observed an effect on the profilin protein. For 48 h of treatment with 5 mM, we observed only a slight decrease for thymosin β4 and profilin (Figure 5A,B).

### Cell localization of actin-binding proteins: thymosin β4, gelsolin and profilin

**Figure 6:**
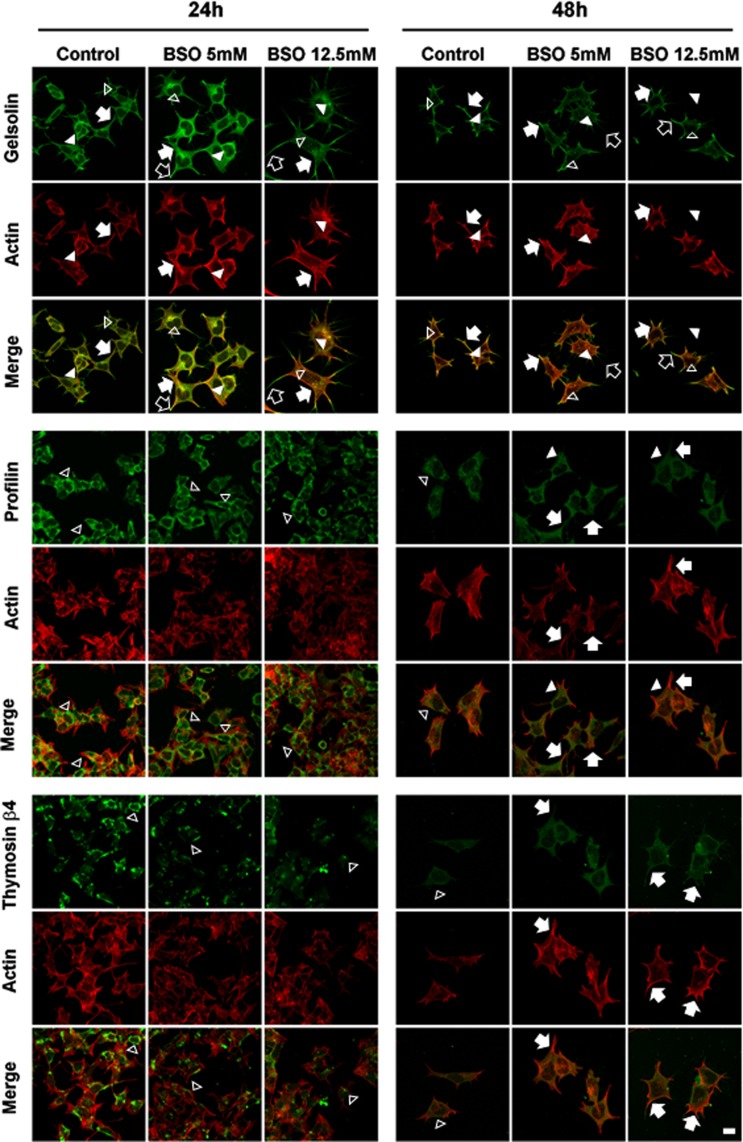
Cell distribution of the actin-binding proteins gelsolin, profilin and thymosin β4. Gelsolin was stained with mouse anti-Gelsolin/FITC-goat anti-mouse (green), Profilin was stained with rabbit anti-Profilin/FITC-goat anti-rabbit (green), Thymosin β4 was stained with goat anti-Thymosin β4/FITC-rabbit anti-goat (green) and actin filaments were stained with Alexa Fluor® phalloidin (red), 40X. Arrows: lamellipodia; arrowheads: filopodia; empty arrow: cone shape; empty arrowheads: cytoplasmic projections and dot-shaped structures.

We performed an immunofluorescence analysis of thymosin β4, gelsolin and profilin for a comparison with the actin filament distributions (Figure 6). After 24 h treatment, gelsolin was widely distributed in both the cytoplasm and cytoplasmic projections of the control cells (either lamellipodia or filopodia). In the cytoplasm, we found gelsolin both free (empty arrowhead) and somewhat co-localized with actin filaments (arrowhead), whereas it co-localized with actin filaments when near the membrane and cytoplasmic projections (arrow). After the 5 and 12.5 mM BSO treatments, gelsolin was found free in the cytoplasm (empty arrowhead) and co-localized with actin filaments, similar to the control cells (arrowhead). However, near the cell membrane and in the cytoplasmic projections, gelsolin was strongly co-localized with the actin filaments. This co-localization was more evident in the cytoplasmic projections (arrow). Notably, in the BSO treatments, especially with 12.5 mM, free gelsolin was present at the distal ends of the projections (empty arrow). After 48 h in the control cells, gelsolin was distributed in both the cytoplasm and the cytoplasmic projections; some cytoplasmic gelsolin was free (empty arrowhead), whereas it was found co-localized with actin filaments near the cell membrane (arrowhead) and in the cytoplasmic projections (arrow). After 48 h in the 5 and 12.5 mM BSO treatments, gelsolin was distributed in the cytoplasm (empty arrowhead), in the vicinity of the cell membrane (arrowhead) and in the cytoplasmic projections (arrow). Its co-localization with actin filaments was more evident in the cytoplasmic projections, where it strongly co-localized with the actin filaments (arrow). Similar to the 24 h treatments, free gelsolin was present at the distal end of the cytoplasmic projections or filopodia (empty arrow).

After 24 h of treatments, profilin showed a similar distribution in both the control cells and the cells treated with 5 and 12.5 mM BSO. Profilin was confined in the cytoplasm located away from the cell membrane and did not co-localize with the actin filaments (empty arrowhead). After 48 h of treatment, the control cells exhibited the same behavior, with the protein confined to the cytoplasm without reaching the region of the cytoplasmic membrane (empty arrowhead). In contrast, the distribution of the protein became more widespread in the cells treated with 5 and 12.5 mM BSO (arrowhead), even reaching the cell membrane and co-localizing with actin filaments (arrow).

Thymosin β4 distribution in the control cells was restricted to the cytoplasm, and no co-localization with actin filaments was observed (empty arrowhead), after 24 h of 5 and 12.5 mM BSO treatments, Thymosin β4 also showed the same distribution in the cytoplasm as the control cells (empty arrowhead). After 48 h of treatment, thymosin β4 in the control cells was distributed in the cytoplasm (empty arrowhead). However, after treatment with 5 and 12.5 mM, the distribution of thymosin β4 reached the cytoplasmic projections and was co-localized with the actin filaments (arrow).

## Discussion

Considering the essential role of glutathione in physiological cell functions and from the predictions obtained from genomic analysis in GCS-2 cells, unable to synthesize GSH (Rojas *et al.*, 2003; Valverde *et al.*, 2006), it was shown that the remodeling of the actin cytoskeleton and accessory pathways are regulated by the tripeptide. To test this hypothesis we evaluated the role of GSH in regulating the actin cytoskeleton in neuroblastoma MSN cells. We assessed the 5 and 12.5 mM BSO concentrations, which depleted GSH by 70% but did not affect cell viability at 24 or 48 h. These results show that MSN cells are more sensitive to GSH depletion than other tissues (such as those in the kidney or liver) and agree with previous research (Stabel-Burow *et al.*, 1997).

BSO treatment triggers a consistent but not complete GSH depletion. According to the literature, the GSH depletion was partial, suggesting that two pools of GSH are present in MSN cells: one that is easily depleted by BSO and another that is more resistant to depletion (Chen *et al.*, 2005). This finding supported the existence of two sources of GSH.

Having demonstrated the decrease in intracellular GSH levels, we evaluated the redox state in MSN cells after treatment with BSO by measuring the ROS levels and end products of lipid peroxidation. Notably, our aim was to decrease the intracellular GSH level without reaching oxidative stress to determine the role of GSH in regulating the actin cytoskeleton because oxidative stress has already been demonstrated to alter the actin cytoskeleton (Fiaschi *et al.*, 2006; Johansson and Lumberg, 2007; Pierozan *et al.*, 2016).

No changes in the ROS level were found compared to the control condition, suggesting that the depletion of the intracellular GSH level did not induce ROS generation (Table 3). Additionally, we measured lipid peroxidation end products and found that MSN cells treated with 5 and 12.5 mM BSO for 24 h were unable to generate lipid peroxidation end products; in contrast, a slight increase was observed after the 48 h treatment with 12.5 mM BSO. To ensure that the loss of GSH did not induce oxidative stress, we conducted a multiple linear regression analysis to evaluate the influence of ROS and lipid peroxidation due to GSH depletion by BSO treatment on cell viability (Table 4). The analysis showed that neither ROS nor lipid peroxidation appeared to be necessary to predict cell viability.

Thus, the detected ROS and lipid peroxidation levels did not affect cell viability and non-oxidative stress generation could be inferred. This agrees with Franco and Cidioswki (2009), as well as Han *et al.* (2007), who support the notion of a direct role for GSH independent from oxidative stress. ROS overload may simply be an epiphenomenon associated with the depletion of GSH.

GSC-2 microarray data and MSN gene and protein expression results confirms that the lack of intracellular GSH modulate the gene expression of thymosin β4, gelsolin and profilin. We observed an important decrease in the expression of these genes. However, the microarray data indicated only the down-regulation of thymosin β4 and profilin, while gelsolin was up-regulated. This discrepancy could be due to different cell types used in each study (blastocysts and neuroblasts), or because blastocyst cells were unable to synthesize GSH, with approximately 2% of the normal amount of GSH. Our study never reached the levels of GSH inhibition obtained by previous research (Shi *et al.*, 2000). The same behavior was observed at the protein level, where we observed only a discrete 20% reduction at 24 h that was absent at 48 h.

The confocal microscopy images showed drastic morphological changes in MSN cells when GSH was depleted by BSO treatment. We stained actin filaments and microtubules (another important component of the cytoskeleton) to visualize the distribution of the actin cytoskeleton and, therefore, the cell shape compared to the microtubule distribution. Actin filaments are abundant beneath the plasma membrane, where they form a network and extend throughout the cytoplasm. The control cells showed a characteristic cell shape and were polarized: a lamellipodium and some filopodia were present at one end, while the far end had a cone shape and focal adhesions were present.

The BSO treatments resulted in the loss of the characteristic cell shape and changes in cell polarity. Large cytoplasmic projections were observed along the cell surface, both lamellipodia and filopodia were present and focal adhesions with a re-localization of thymosin β, gelsolin and profilin proteins were detected. Moreover, the cells contained a neurite-like structure. In this case, the structure resembled an axon due to the neuronal origin of the MSN cells. These observations agree with our previous results (Ramos-Espinosa *et al.*, 2012) and with Mollinari *et al.* (2009), which showed enhanced neurite outgrowth accompanied by increased focal adhesions due to the down-regulation of thymosin β4.

To visualize the changes described above, we used the image processing package Fiji to analyze 25 representative images of the cell shapes in the control and BSO-treated cells. The predominant cell shapes in the control conditions were polarized with a lamellipodium, with filopodia present at one end, whereas the far end had a cone shape. However, the different BSO treatments resulted in the constant and evident loss of the characteristic cell shape, dramatic polarity changes, more evident cytoplasmic projections, the presence of either lamellipodia or filopodia and in some cases filopodia that resembled an axon that gave the cell a neuron-like cell shape. The drastic changes induced by the BSO treatments went beyond the ultrastructure of the actin cytoskeleton. Notably, we found that GSH depletion could alter the regulation of actin cytoskeleton dynamics, thereby causing the formation of neurites in cultured MSN cells. Notably, these changes did not occur in an undifferentiated cell culture, eliminating a possible effect of BSO per se. The neurite formation process is regulated in complex ways. Its growth and guidance depend on well-coordinated cytoskeletal dynamics and occur in conjunction with differentiation and plasticity processes (Wilson *et al.*, 2016). Thus, these results suggest that a decrease in the intracellular GSH content can affect processes involved in the signaling pathway that regulates neurite growth (Bradke and Dotti, 2000a,b; Luo, 2002; Madduri *et al.*, 2009; Wilson *et al.*, 2016).

In conclusion, GSH depletion produced a downregulation of the actin binding proteins profilin, thymosin β4 and gelsolin after 24 h BSO treatments. This down-regulation appears to be sufficient to trigger important changes in their localization and cellular shape in a non-oxidative stress-dependent manner. These results are relevant because exposures to xenobiotics could decrease the levels of GSH and could represent a cofactor that triggers changes in the cytoskeleton to facilitate the acquisition of several disease hallmarks including those related to cancer and neurodegenerative diseases.
